# Therapeutic potential of a rhein-loaded self-nano-emulsifying drug delivery system in ameliorating LPS-induced depression: mechanistic insights and behavioral outcomes

**DOI:** 10.3389/fphar.2025.1577080

**Published:** 2025-05-23

**Authors:** Sachin More, Md Abdur Rashid, Prashant Kumar Tiwari, Rohini Kharwade, Yahya Alhamhoom, Turky Omar Asar, Mohammed Kaleem, Nilesh Mahajan, Ajay Pise, Kishor Danao, Sanjay Kumar

**Affiliations:** ^1^ Department of Pharmacology, Dadasaheb Balpande College of Pharmacy, Rashtrasant Tukadoji Maharaj Nagpur University, Nagpur, Maharashtra, India; ^2^ Department of Pharmaceutics, College of Pharmacy, King Khalid University, Abha, Saudi Arabia; ^3^ Department of Life Sciences, Sharda School of Bio-Science and Technology, Sharda University, Greater Noida, Uttar pradesh, India; ^4^ Department of Pharmaceutics, Dadasaheb Balpande College of Pharmacy, Rashtrasant Tukadoji Maharaj Nagpur University, Nagpur, Maharashtra, India; ^5^ Department of Biology, College of Science and Arts at Alkamil, University of Jeddah, Jeddah, Saudi Arabia; ^6^ Department of Regulatory Affairs Dadasaheb Balpande College of Pharmacy, Rashtrasant Tukadoji Maharaj Nagpur University, Nagpur, Maharashtra, India

**Keywords:** rhein, self-nano-emulsified drug delivery system, neuroinflammation, depression, cytokines, lipopolysaccharides

## Abstract

Depression is a multifaceted disorder caused by neuroinflammation, which is mainly demarcated by a significant increase in proinflammatory cytokines, including interleukin-1β (IL-1β), interleukin-6 (IL-6), and tumor necrosis factor-α (TNF-α). Conventional treatments for depression typically focus on neurotransmitter theories and may lead to several undesirable side effects. Therefore, it is essential to identify innovative active compounds of herbal origin that can target proinflammatory cytokines to reduce neuroinflammation while minimizing side effects. Rhein has demonstrated considerable therapeutic efficacy in various neurological conditions; however, its mechanistic insights regarding antidepressant effects remain unclear. An *in silico* study of rhein against the putative target enzyme of depression showed prominent binding with neuroinflammatory proteins 1ALU, 2AZ5, and 5R88, achieving docking scores −5.84 kcal/mol, −5.23 kcal/mol, and −5.243 kcal/mol, respectively. However, the poor absorption of rhein limited its therapeutic efficacy. To address this issue, a rhein-loaded self-nano-emulsifying drug delivery system (R-SNEDDS) was developed and evaluated for its therapeutic effects in preventing a lipopolysaccharide-induced depression model in rats. The study found that intraperitoneal administration of R-SNEDDS (at doses of 50 mg/kg and 100 mg/kg rhein, i.p.) and duloxetine (as a positive control at 20 mg/kg) over three consecutive days reversed unusual depressive behaviors. Notably, the R-SNEDDS (100 mg/kg rhein, i.p.) significantly reduced levels of the proinflammatory cytokines IL-1β (30.91 ± 0.906), IL-6 (133.9 ± 2.232), and TNF-α (26.93 ± 1.807) compared to the lipopolysaccharide-induced group. These findings demonstrate that R-SNEDDS possesses anti-neuroinflammatory properties and could be promising for depression therapy.

## 1 Introduction

Depression is a type of mental disorder characterized by aberrant feelings, sadness, and mental and physical impairments that interfere with daily activities throughout the lifespan and may lead to suicidal thoughts (Bai et al., 2024). The underlying causes of depression are multifaceted, including genetic, environmental, and biological factors (Burstein and Doron, 2018; Kharwade and More, 2021). According to evidence, increasing levels of inflammatory markers such as IL-1β, IL-6, and TNF-α may be associated with depression. These indicators change neurotransmitter metabolism, neuroendocrine system function, and neuroplasticity and perform a vital role in the pathophysiology of depression (More S. et al., 2025). Bosker et al. observed that the blood and cerebrospinal levels of IL-6 and TNF-α were significantly increased in depressed patients older than 60 years. They also reported that IL-1β was increased in both peripheral blood circulation and cerebrospinal fluid in patients exposed to stress and accompanied by symptoms of depression compared to non-depressed women (Salokangas et al., 2002; Bosker et al., 2004).

Traditional antidepressant therapy includes serotonin reuptake inhibitors, serotonin stimulators, monoamine oxidase inhibitors, norepinephrine reuptake inhibitors, and monoamine reuptake blockers (Ahmad et al., 2016a; Fanelli et al., 2021). Many therapeutic molecules target the monoamine theory of depression. However, major depressive disorder often does not respond to conventional antidepressants due to elevated levels of inflammatory markers, leading to various side effects (Duman, 2004; Massart et al., 2012). It is essential to investigate complementary prophylactic antidepressants that target inflammatory cytokines to manage depression-like behavior in severely ill patients who have not responded to monoamine therapy.

Recently, many phytoconstituents have proven their role in neuroprotection by modifying various neurotransmitters, regulating neurogenesis, and reducing oxidative stress. Anthraquinones have exhibited the best neuroprotective effect (Naoi et al., 2019). Rhein is an anthraquinone phytoconstituent with proven anti-inflammatory, antioxidant, and neuroprotective activities (Li et al., 2019). In addition, rhein is the only anthraquinone that crosses the blood–brain barrier, but its therapeutic effectiveness was restricted due to its poor solubility and low bioavailability, which may alter its therapeutic efficacy (Kharwade et al., 2022). Therefore, there is a need to formulate rhein into a novel drug delivery system. In this regard, a solid self-nano-emulsifying drug delivery system (SNEDDS) can improve the dissolution rate, solubility, release rate, and intraperitoneal bioavailability (Laddha et al., 2014; Baloch et al., 2019; Kharwade et al., 2022). The current study revealed that rhein-loaded self-nano-emulsifying drug delivery (R-SNEDDS) formulation could be a potential strategy for improving oral bioavailability to deliver the drug to central nervous system (CNS) disorders (More S. M. et al., 2025). Bi et al. (2020) observed that rhein inhibited the *in vitro* equiaxial stretch-induced neuron pyroptosis and lipopolysaccharide (LPS) release and downregulated the proinflammatory factors. However, mechanistic insights concerning antidepressant action were not revealed. Therefore, the current investigation aims to accomplish the molecular docking of rhein to reveal the potential binding of rhein to target protein of proinflammatory cytokines.

LPS is the main content of the outer membrane of Gram-negative bacteria *Escherichia coli*. It is a commonly used proinflammatory endotoxin that can trigger immune cells in the brain; that is, microglial activation leads to the activation of intense immunological reactions (Koch et al., 1992; Ohgi et al., 2013). These reactions release extra proinflammatory cytokines such as IL-1β, IL-6, and TNF-α, easily penetrating the blood–brain barrier and contributing to neuroinflammation and neural injury. According to the literature, LPS also activates the hypothalamic–pituitary–adrenal (HPA) axis and initiates the production of the stress hormone cortisol. It is also associated with increased IL-6 concentration in blood circulation (Rein et al., 2019; Kharwade et al., 2024). LPS-induced models of depression include the studies by Yin et al. (2023) and Domingues et al. (2018). Scientists observed that a single intraperitoneal dose of LPS at 0.5 mg/kg and 0.83 mg/kg leads to an increase in neuro-inflammatory cytokines and oxidative stress hormones. Behavioral assessments revealed increased immobility time during the tail suspension test and a reduced preference for sucrose. These findings confirmed that LPS triggers inflammation in animals, establishing LPS-administered rats as a model for neuroinflammation related to depression (Domingues et al., 2018; Yin et al., 2023).

In the present study, we conducted an *in silico* analysis of rhein and identified target proteins of cytokines. This was followed by the formulation of a rhein-loaded self-nanoemulsifying drug delivery system (R-SNEDDS). We then evaluated the R-SNEDDS for its solubility, intraperitoneal bioavailability, and prophylactic antidepressant effects (More S. M. et al., 2025).

Using an LPS-induced rat model, we analyzed the levels of proinflammatory cytokines using the ELISA method. Additionally, we assessed depression-like behavior through various tests, including the tail suspension test (TST), open-field test (OFT), forced swim test (FST), and sucrose preference test (SPT).

## 2 Materials and methods

### 2.1 Chemicals

All the reagents used in this study were of high analytical-grade quality. Rhein was procured from TCI Chemicals, Japan. LPS from *E. coli,* serotype 0127: B8, was purchased from Sigma-Aldrich, United States. Alembic Pharma Gujarat, India, provided a gift sample of duloxetine. An immunoassay kit of IL-1β, IL-6, and TNF-α was procured from Elabscience Ltd., United States, with sensitivities for IL-6 = 3.3 pg/mL; IL-1β = 18.75 pg/mL; and TNF-α = 9.38 pg/mL. Sucrose was purchased from Sisco Research Laboratories Pvt. Ltd., Mumbai, India.

### 2.2 Experimental animals

A total of sixty (n = 60) healthy Sprague–Dawley (SD) rats with an age of 9–12 weeks and 230–250 g of weight were sanctioned and approved by the Institutional Animal Ethics Committee (DBCOP/IAEC/1426/2022–23/P11). All animals were kept under standard conditions according to Committee for Control and Supervision of Experiments on Animals (CCSEA) guidelines and institutional policies on animal experimental handling guidelines. Rats were maintained in standard polypropylene cages (4 rats/cage) under room temperature, humidity (60%−70%), and 12-h dark/light cycles. The diets were available *ad libitum* (Kaleem and Haque, 2015).

### 2.3 Methods

#### 2.3.1 Collection of ligands and receptors

**FIGURE 1:**
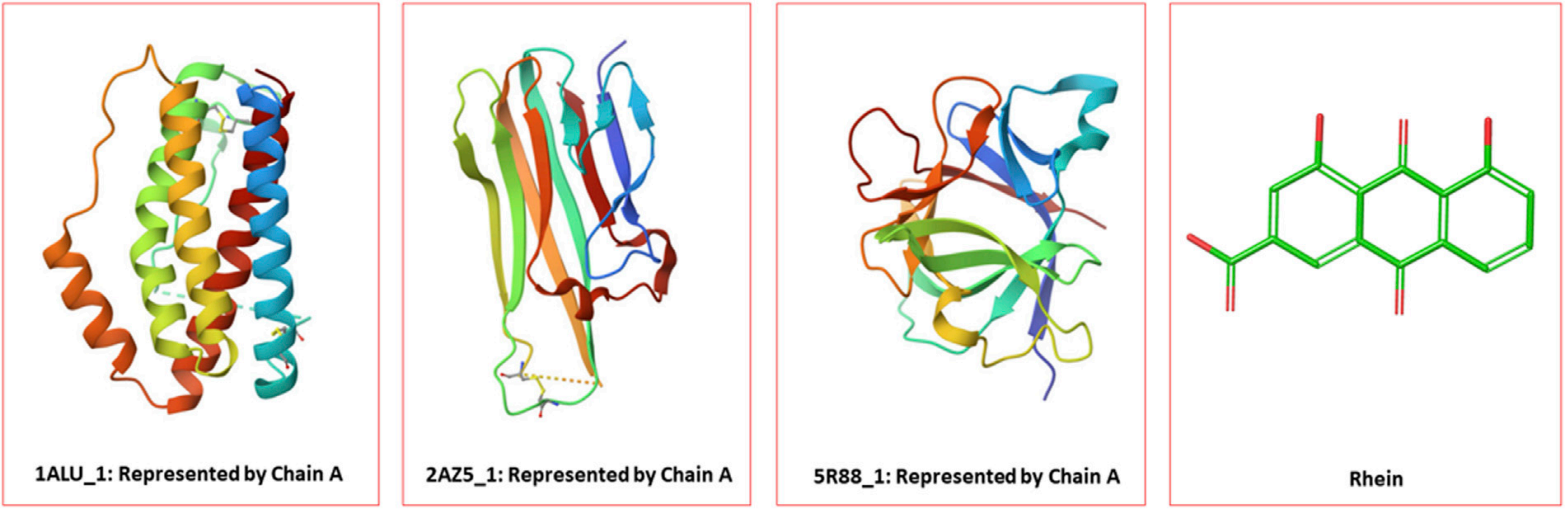
1ALU, 2AZ5, and 5R88 proteins and rhein ligands.

The crystal structures of interleukin-6 (IL-6), tumor necrosis factor-alpha (TNF-α) receptor, and interleukin-1 beta (IL-1β) proteins with the respective PDB IDs 1ALU, 2AZ5, and 5R88 were retrieved from the RCSB Protein Data Bank (https://www.rcsb.org/). The crystal structure of interleukin-6 (IL-6) (1ALU) was determined at a resolution of 1.90 Å, with a total molecular mass of 21.67 kDa. The structure consists of protein chain A and a co-crystallized smaller compound, L-(+)-tartaric acid, along with a sulfate ion. The structure of TNF-α (2AZ5) was determined at a resolution of 2.10 A and a molecular mass is 66.74 kDa. The structure consists of chain A, co-crystallized 6,7-dimethyl-3-[(methyl{2-[methyl({1-[3-(trifluoromethyl)phenyl]-1H-indol-3-yl}methyl)amino]ethyl}amino)methyl]-4H-chromen-4-one. The crystal structure of IL-1β (5R88) with (2∼{S})-∼{N}-(4-aminocarbonyl phenyl)oxolane-2-carboxamide has been determined at a high resolution of 1.48 Å. Rhein compounds were docked against these proteins. The same co-crystal ligands have been utilized for the reference compounds. Rhein is noted to have hepatoprotective, anticancer, anti-inflammatory, and nephroprotective properties (PubChem CID 10168). Rhein is found in many plants, including *Rheum palmatum*, and is a metabolite of rhein anthrone and senna glycosides. The rhein structure was downloaded from ChemSpider (molecular formula: C_15_H_8_O_6_, ChemSpider ID: 9762) for docking against these proteins (https://www.chemspider.com/). (Figure 1 shows 1ALU, 2AZ5, and 5R88 proteins and rhein ligands).

#### 2.3.2 Optimization of proteins and ligands

IL-6, TNF-α, and IL-1β proteins were optimized for molecular docking studies, and their refinement was performed using the Protein Preparation Wizard in Maestro (version 9.0.111) (Bathula, 2022). The Protein Preparation Wizard was utilized for protein preparation and refinement, including the removal of water molecules, the addition of missing hydrogen atoms, and the assignment of the correct bond order using the OPLS4 force field. To obtain a stable structure, energy minimization was performed until the root mean square deviation (RMSD) value reached 0.30 Å. Additionally, the rhein ligand molecules were refined using the LigPrep module of the Schrödinger suite (OPLS4 force field), ensuring accurate structural optimization for molecular docking studies. Epik was used to generate different stereochemical forms as well as different ionic and tautomeric states of the ligand at pH 7.0 ± 2.0. Additionally, five low-energy ring conformations were prepared, ensuring a comprehensive structural representation for molecular docking and further computational analysis (Madhavi Sastry et al., 2013).

#### 2.3.3 Identification of binding sites

Structure-based drug design primarily focuses on identifying specific binding sites on target proteins to develop drugs that can bind to these sites. This enables the drug to block or inhibit the function of proteins, ultimately stopping or slowing cell progression. To facilitate drug binding to the active sites of IL-6, TNF-α, and IL-1β, the active sites of the proteins were identified at precise locations corresponding to the co-crystal ligands. A three-dimensional grid box generated around the co-crystal ligand using the receptor grid generation program in the Glide tool provided the basis for accurate molecular docking studies (Alogheli et al., 2017).

#### 2.3.4 Molecular docking using Glide

Schrödinger’s Glide module was used for molecular docking studies to predict the binding interactions between selected ligands and target receptors. The OPLS4 force field was utilized for energy minimization, and extra precision (XP) docking mode was used to enhance accuracy. The OPLS4 force field was used for energy minimization. Docking calculations followed the default convergence criterion, which ensured energy minimization until the gradient reached 0.01 kcal/mol Å.

#### 2.3.5 Validation of post-docking result and RMSD calculation

Post-docking validation was performed by calculating the RMSD value between the co-crystallized ligand and the best-docked pose. RMSD values were calculated using PyMOL software, where ligand structures were aligned, and the deviation was measured to assess docking accuracy. RMSD values less than 2.0 Å were considered reliable.

#### 2.3.6 Binding free energy calculation

Molecules obtained from Extra Precision (XP) docking were selected to evaluate the binding affinity of the ligands with the protein. Prime MM-GBSA calculations for this analysis were performed using the OPLS4 force field. The binding free energy quantifies the affinity between the ligand molecules and the protein. Calculations were performed on specific binding poses of the protein–ligand complex to evaluate its stability and interaction strength (Bathula, 2022) (Equation 1).

$$∆G_{\text{Binding}}=∆G_{\text{Complex}}-∆G_{\text{protien}}+∆G_{\text{Ligand}},$$ (1)

where ΔG_Binding_ is minimized binding free energy and ΔG _complex_, ΔG _protein_, and ΔG _ligand_ denote the free energy of the protein–inhibitor complex, protein, and inhibitor, respectively.

#### 2.3.7 Drug-likeness and ADME properties

The pharmacokinetic properties and the drug-like characteristics of the docked natural compound rhein were evaluated using absorption, distribution, metabolism, and excretion (ADME) analysis and SwissADME online tool. This analysis provides valuable insights into its suitability for further drug development and optimization (Daina et al., 2017). This online tool evaluates various physical properties of the ligand, including water solubility, kinetics, drug affinity, medicinal chemistry, lipophilicity, and physicochemical properties. This evaluation helps determine whether compounds follow Lipinski’s rule of five (molecular weight <500; QPlogPo/w < 5; DonorHB <5; acptHB <10) (Kumar Tiwari et al., 2024).

#### 2.3.8 Grouping

The 60 SD rats were divided into six groups; each group comprised 10 rats (n = 10). Treatment for each group was as follows.

(a) Group I received equivolume sterile, endotoxin-free 0.9% w/v saline solution.
(b) Group II received LPS dissolved in sterile, endotoxin-free 0.9% w/v saline (0.83 mg/kg).
(c) Group III received LPS and SNEDDS without rhein.
(d) Group IV received LPS and duloxetine as standard (20 mg/kg, i.p.).
(e) Groups V and VI received low (50 mg/kg, i.p.) and high (100 mg/kg) R-SNEDDS doses, respectively (More S. M. et al., 2025).

The LPS and duloxetine doses were selected following a previous study (Álvarez-González et al., 2021).

#### 2.3.9 Experimental protocol

Animals from groups III, IV, V, and VI were pretreated with the respective drug by an intraperitoneal (i.p.) route at 10:00 a.m. daily for three consecutive days. On day three, all animals except Group I received 0.83 mg/kg (i.p.) of lipopolysaccharide 30 min after treatment. Group I animals received an equivolume of sterile, endotoxin-free 0.9% w/v saline solution.

All animals were monitored for any sign of sickness after LPS administration. All animals were analyzed for behavioral assessment 24 h after LPS and saline administration using an OFT and TST [n = 5], as well as a SPT and a FST [n = 5].

**FIGURE 2:**
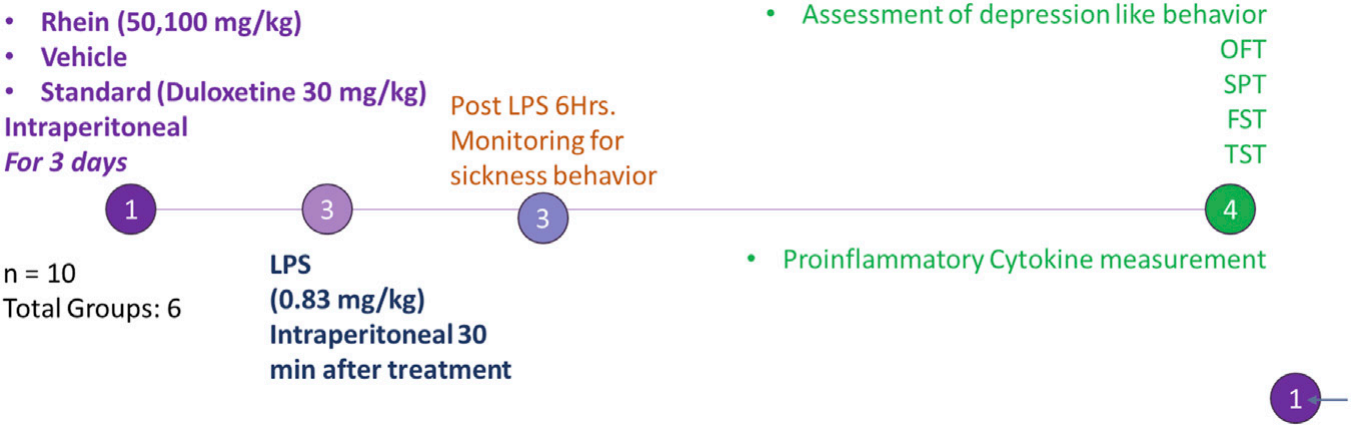
Schematic representation of experimental protocol.

After the behavioral assessment, five animals from each group were euthanized, and the brain was isolated and homogenized in phosphate-buffered saline (PBS) solution for biochemical estimation of proinflammatory cytokines by the ELISA method. All experimental procedures were performed as per the guidelines of CCSEA. A schematic representation of the experimental design is depicted in Figure 2.

### 2.4 Behavioral assessment

#### 2.4.1 Tail suspension test

The well-recommended tail suspension test is a behavior experiment used to assess depression-like behavior in animals. It measures the stress and denotes the distressed state of animal behavior. In this test, the rat was hung on 50 cm of the crossbar, and the last 4 cm of the tail was fixed with tape. Initially, rats struggle to overcome the abnormal positions and become immobile after some time, which was recorded within 6 min. The first 1 min is considered an acclimatization period, and a reading of the last 5 min is considered for assessment (Moreau et al., 2009).

#### 2.4.2 Forced swim test

A forced swim test is another widely accepted model to determine depressive behavior in animals. The rats were forced to swim in a 30-cm-diameter cylindrical glass jar filled with water at 24 ± 2^o^C. Rats should be able to escape and touch the bottom of the jar. To avoid swimming stress, rats were subjected to a 15-min acclimatization in a jar with water. During the final 6-min assessment, immobility time (the total time when animals stop struggling to escape and remain floating), was recorded. (Zeldetz et al., 2018).

#### 2.4.3 Open field test

The open-field test is a behavioral assessment experiment that measures neurobiological animal activity, anxiety, and inclination to explore a controlled condition. The apparatus comprised a wooden box of 100 cm × 100 cm with a boundary wall height of 50 cm. It was equally divided into 16 equal squares with lines marked on the floor. The rat was placed at the central square, and the number of squares crossed and the number of rearing instances were evaluated for 5 min. The apparatus was cleaned with 5% ethanol before starting the study and between each rat (Li et al., 2014).

#### 2.4.4 Sucrose preference test

Anhedonia, a milestone indication of depression, was assessed by a sucrose preference behavioral experiment. It was carried out 24 h after LPS administration. The experiment was performed as described by Willner (1997) with minor modifications. Before starting the experiment, rats acclimate with sucrose solution (1% w/v) for 24 h. In the next 24 h, one of the sucrose solution bottles was replaced with tap water, followed by withdrawal of water and food for the next 12 h.

During the test, the rats had free access to 100 mL sucrose solution and 100 mL water for 24 h. The consumption of sucrose and water was noted (Liu et al., 2018). The sucrose preference was determined following Equation 2.

$$\text{The sucrose preference }\left(\%\right)=\frac{\text{Sucrose}\;\text{consumption}}{\text{Sucrose}\;\text{consumption}+\text{water}\;\text{consumption}}\times 100.$$ (2)

#### 2.4.5 Biochemical estimation (inflammatory cytokine estimation)

Neurochemical estimation and level of proinflammatory cytokines were determined by the ELISA technique. The study comprised the removal of the whole brain after PBS perfusion via the heart under anesthesia. It was homogenized with protease and phosphatase inhibitors in PBS buffer and cold centrifuged under 10,000 rpm for 5 min (Remi, CM12 plus, India). The supernatant was collected and immediately stored at −40°C (Remi, RQVD200 plus, deep freezer, India) until the ELISA assay (Barua et al., 2018). The ELISA for TNF-α, IL-1β, and IL-6 was per the procedure mentioned in the kit (Elabscience). The absorbance was recorded at 450 nm in a microplate reader (Thermo Fisher Scientific Varioskan LUX multimode microplate reader, New York).

#### 2.4.6 Histopathological assessment

The animals were euthanized by cervical decapitation to eliminate the potential influence of anesthesia on the brain. The rat brains were dissected along the sagittal plane into two parts on a chilled surface. The right part of brain tissue was fixed with 10% formalin and subsequently processed to form blocks. The 3–4-μm-thick sections were prepared and stained with hematoxylin and eosin (H&E) for histopathological analysis, following the protocol described by Bancroft and Gamble (2007) and Ahmad et al. (2016b). The slides were observed in a light microscope.

#### 2.4.7 Data analysis

All data were expressed in the mean ± SEM form. The statistical analysis was performed following a one-way analysis of variance (ANOVA) and *post hoc* Tukey’s multiple comparison test. *p < 0.05, **p < 0.01, ***p < 0.001 were considered statistically significant.

## 3 Results

### 3.1 Docking analysis and binding energies

Molecular docking used the Glide tool for ligand docking to determine whether the rhein molecule binds at the same site as the co-crystallized native ligands of IL-6, TNF-α, and IL-1β. Utilizing the receptor grid generation feature in the Glide tool of the Schrödinger suite, a grid box was drawn at the same location where the native ligand was bound. This was done to obtain optimal docking interactions within the defined binding domain. Based on the previous step, rhein molecules were docked using the standard precision (XP) docking mode that gives more precise conclusions and generates the lowest energy conformers of the ligand, ensuring optimal binding interactions within the previously defined grid box. Molecular docking studies showed that rhein demonstrated stronger binding affinities than the native ligand for the 1ALU, 2AZ5, and 5R88 targets, as assessed by the docking, Glide, and MM-GBSA scoring systems. For 1ALU, rhein demonstrated better binding with a docking score of −5.849, a Glide score of −5.84, and an MM-GBSA binding energy of −38.07 kcal/mol, which was higher than that of the native ligand (docking/Glide score: 4.496; MM-GBSA).

Similarly, rhein displayed strong binding to 2AZ5 with a docking score of −5.232, Glide score of −5.232, and MM-GBSA energy of −31.51 kcal/mol. In contrast, the native ligand of 2AZ5 showed weak docking (−4.5) and Glide (−4.784) scores but a high MM-GBSA stability (−46.04 kcal/mol), indicating divergent binding mechanisms. For 5R88, rhein achieved a docking/Glide score of −5.243 and an MM-GBSA energy of −26 kcal/mol, which surpasses the docking (−4.49) and Glide (−4.79) scores of the native ligand. However, the native ligand displayed greater MM-GBSA stability (−35.6 kcal/mol), highlighting possible differences in interaction strengths or solvation effects. Negative ΔG values suggest the formation of thermodynamically stable complexes (Table 1) (Rajagopal et al., 2019). The RMSD values of 1ALU (1.79 Å), 2AZ5 (3.6 Å), and 5R88 (2.57 Å) were analyzed to assess docking accuracy; among them, the 1ALU complex showed a reliable docking pose, while 5R88 remained within an acceptable range. However, 2AZ5 exhibited a higher deviation, indicating possible flexibility in the binding site. These results suggest that 1ALU and 5R88 show good alignment.

**TABLE 1:** Docking score/Glide score and MM-GBSA binding energy calculated by Glide Maestro and interacting residues in docking with rhein.

Target protein	Docking score		Glide score		MM-GBSA binding energy (kcal/mol)		Interacting residues in docking with rhein
	Rhein	Reference compound	Rhein	Reference compound	Rhein	Reference compound	
IL-6	−5.849	−4.496	−5.84	−4.496	−38.07	−8.63	Arg30, Leu33, Asp34, Gln175, Leu178, Arg179, and Arg182
TNF-α	−5.232	−4.5	−5.232	−4.784	−31.51	−46.04	His15, Leu33, Asp34, Leu57, Tyr59, Gln61, Tyr119, Tyr151, Ile155, Gln175, Leu178, Arg179, and Arg182
IL-1β	−5.243	−4.49	−5.243	−4.79	−26.00	−35.6	Lys74, Tyr24, Leu82, Val132, Thr79, Gln81, Leu80, Pro131, and Phe133

### 3.2 Molecular docking analysis of rhein against IL-6: insights into binding stability

**FIGURE 3:**
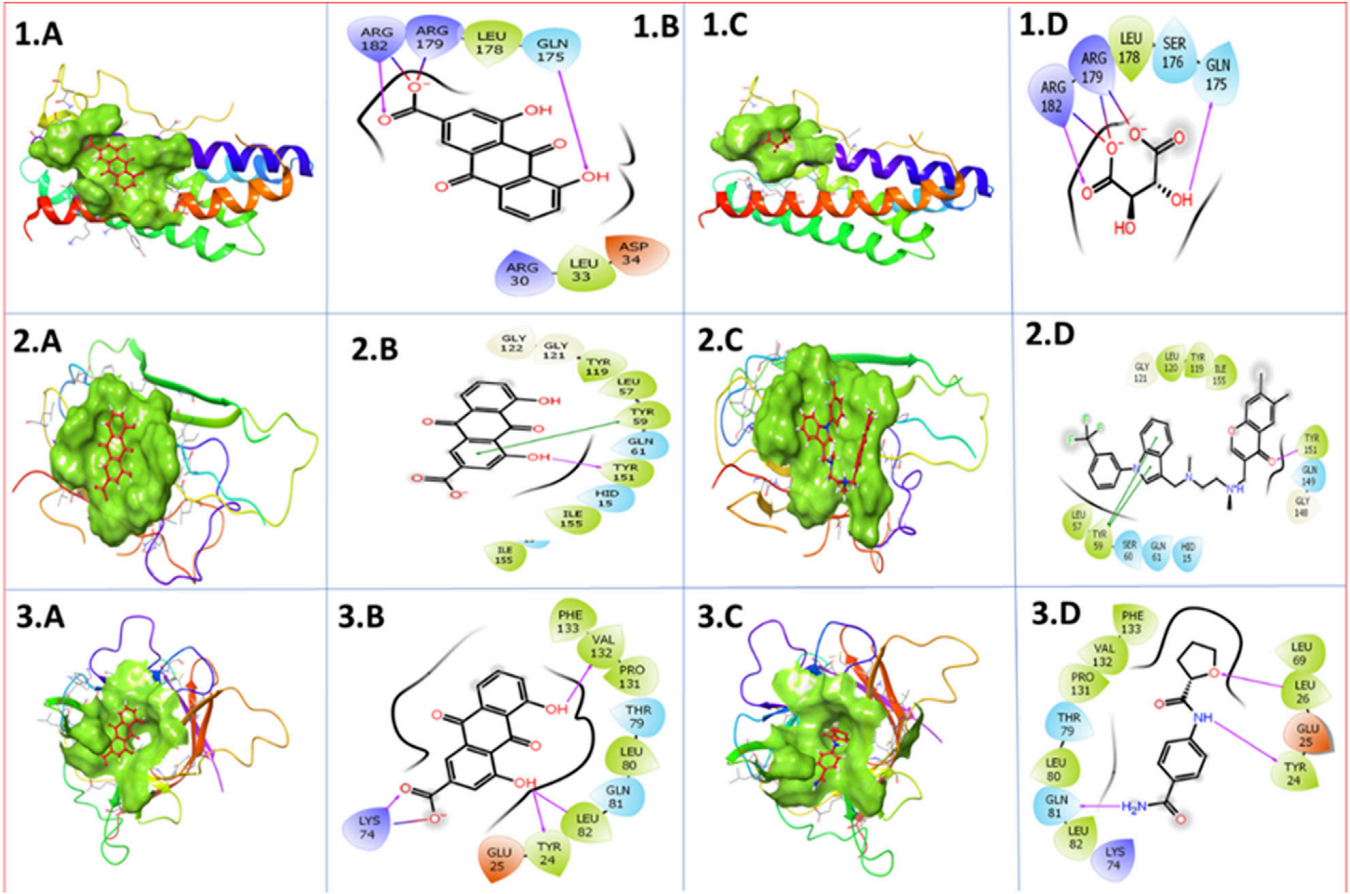
3D and 2D docked complexes of the selected phytochemical-derived natural compounds rhein with IL-6 (1A, 1B) and reference compounds with IL-6 (1C, 1D). Rhein with TNF-α (2A, 2B) and reference compounds with TNF-α (2C, 2D). Rhein with IL-6 (3A, 3B) and reference compounds with IL-1β (3C, 3D). In two-dimensional structures: H-bond formation (violet), hydrophobic interaction (green), polar residue (blue), negative residue (red), glycine (gray), and salt bridge (red and blue).

The ligand rhein demonstrated multifaceted binding interactions with IL-6 proteins, characterized by a combination of polar, hydrophobic, ionic, and hydrogen-bonding forces. The major hydrogen bonds involve residues Arg182 and Gln175, with Gln175 also participating in polar interactions, while hydrophobic interactions with Leu33 and Leu178 further stabilize the binding. In particular, a salt bridge with Arg179 and Arg182 and ionic interactions Arg30 and Asp 34 contribute to the stability of the complex [Figure 3 (1.A,1.B)]. The reference compound shares almost identical binding profiles with the reference ligand, including important residues such as Arg182, Gln175, Leu33, Leu178, Arg179, and Arg182 [Figure 3 (1.C,1.D)], suggesting a conserved mechanism of interaction. These findings highlight the ability of the ligand to engage diverse residues through multiple interaction types, emphasizing its potential for targeted binding in IL-6.

### 3.3 Molecular docking analysis of rhein against TNF-α: insights into binding stability

Molecular docking analysis shows that rhein exhibits strong and stable binding interactions with human TNF-α. This strong interaction is facilitated by a combination of hydrogen bonding (Tyr151), hydrophobic contacts (Leu57, Tyr59, Tyr119, Tyr151, and Ile155), polar interactions (His15, Gln61), and ionic interactions (His15) [Figure 1 (2.A, 2.B)]. Notably, rhein shares key binding residues (Tyr119, Ile155, Tyr151, Leu57, Tyr59, Gln61, and His15) with the reference compounds, suggesting a conserved mechanism of TNF-α inhibition Figure 3 (2.C,2.D).

### 3.4 Molecular docking analysis of rhein against IL-1β: insights into binding stability

A strong interaction was observed between IL-1β and the compound rhein. The stability of the complex is reinforced by multiple interaction types, including hydrogen bonding (Lys74, Tyr24, Leu82, and Val132), polar contacts (Thr79 and Gln81), and hydrophobic interactions (Tyr24, Leu82, Leu80, Pro131, Val132, and Phe133). Lys74 is also involved in a salt bridge and ionic interactions [Figure 3 (3.A,3.B)]. Notably, key residues such as Val132, Pro131, Thr79, Leu80, Gln81, Leu82, and Lys74 are shared between rhein and the reference compound [Figure 3 (3.C,3.D)], suggesting a conserved binding mechanism important for IL-1β inhibition.

### 3.5 ADME analysis

Rhein, a natural phytochemical compound, exhibits promising physicochemical and biochemical properties. The molecular weight of rhein is 284.22 g/mol. ADME results showed no violations of Lipinski’s rule of five, indicating a topological polar surface area (TPSA) within the acceptable range for cell membrane permeability and favorable oral bioavailability. The ADME properties of rhein are presented in Table 2.

**TABLE 2:** ADME properties of rhein.

S. no.	Property	Value		Interpretation	
1	Molecular weight (MW)	284.22 g/mol		Within Lipinski’s rule (<500 Da)	
2	Topological polar surface area (TPSA)	111.1 Å^2^		Moderate permeability (should be < 140 Å^2^)	
3	LogP (lipophilicity)	1.85		Moderate lipophilicity (ideal range: 0–5)	
4	Water solubility (logS)	−3.12		Moderately soluble	
5	GI absorption	High		Good oral bioavailability	
6	Blood–brain barrier (BBB) permeability	No		Does not cross BBB (CNS safety)	
7	CYP450 inhibition	No major inhibition		Low risk of metabolic drug-drug interactions	
8	Lipinski’s rule violations	0		Drug-like properties satisfied	

Lipinski’s rule of five analysis for rhein					
Molecule	Molecular weight (MW) ≤ 500	H-bond acceptors (≤10)	H-bond donors (≤5)	Partition coefficient (LogP) (≤5)	Lipinski violation
Rhein	284.22 (yes)	6 (yes)	3 (yes)	N/A (N/A)	0

### 3.6 Behavioral assessment

This study aimed to evaluate the efficacy of duloxetine and rhein at 50 mg/kg and 100 mg/kg administered intraperitoneally (i.p.) in normalizing behavioral alterations caused by lipopolysaccharide exposure, a model often used to induce depressive-like behaviors.

#### 3.6.1 Tail suspension test

**FIGURE 4:**
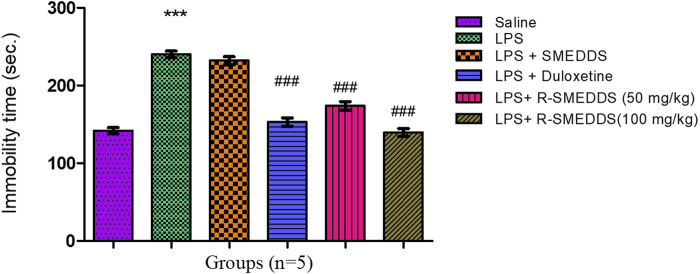
Effects of treatments on immobility time in the tail suspension test in LPS-induced depression in rats. Results are shown as mean ± SEM (n = 05). ***p < 0.001 compared to the control group; #p < 0.05; ###p < 0.001 compared to the LPS group.

LPS-treated rats exhibited significantly increased immobility time (206.0 ± 7.662 s) compared to the saline group (102.0 ± 3.755 s), indicating depressive-like behavior. Administration of both duloxetine and R-SNEDDS (50 mg/kg and 100 mg/kg, i.p.) significantly reduced immobility time (130.8 ± 4.810 s, 179.0 ± 6.285 s, and 135.4 ± 5.085 s, respectively) [F (5,24) = 54.71, *p* < 0.001)] compared to the LPS-only group, with the higher dose of rhein showing more pronounced effects. Immobility time through the TST is outlined in Figure 4.

#### 3.6.2 Forced swim test

**FIGURE 5:**
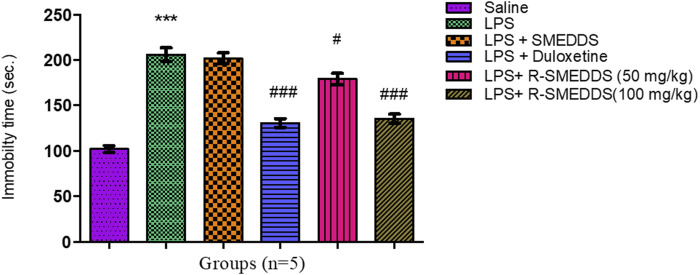
Effects of treatments on immobility time in the forced swim test in LPS-induced depression in rats. Results are shown as mean ± SEM (n = 05). ***p < 0.001 compared to the control group; ###p < 0.001 compared to the LPS group.

Similar to the TST, LPS administration resulted in an increase in immobility time (240.2 ± 4.236 s) in the FST compared to the saline group (141.8 ± 3.967 s). Both duloxetine and rhein reduced immobility (153.0 ± 5.934 s, 173.8 ± 5.687 s, and 139.6 ± 4.915 s) [F (5,24) = 84.55, *p* < 0.001)]. The FST data further support the antidepressant-like effects of rhein, indicating its potential to enhance coping mechanisms in stressful conditions. Duloxetine’s well-known serotonin and norepinephrine reuptake inhibition likely plays a role (Tomaz et al., 2020), while the anti-inflammatory properties of rhein may mitigate neuroinflammation-induced depressive states (Ohgi et al., 2013). The immobility time through FST is outlined in Figure 5.

#### 3.6.3 Open-field test

**FIGURE 6:**
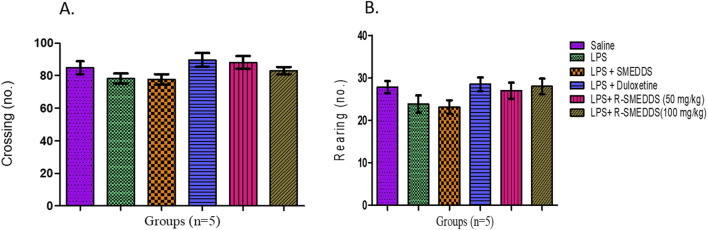
Effects of treatments on **(A)** crossing and **(B)** rearing in the open-field test in LPS-induced depression in rats. Results are shown as mean ± SEM (n = 05).

LPS-treated rats displayed decreased amounts of locomotion (78.17 ± 3.135 lines crossed) and exploratory behavior (23.83 ± 2.040 rearings) compared to the saline group (84.83 ± 3.995; 27.83 ± 1.400). Both duloxetine and rhein (50 mg/kg and 100 mg/kg, i.p.) normalized locomotion (89.67 ± 4.240; 88.17 ± 3.885 and 83.00 ± 2.295) and exploratory behavior (28.50 ± 1.607; 27.00 ± 1.880 and 28.00 ± 1.807). The total numbers of crossings and rearings are shown in Figure 6.

#### 3.6.4 Sucrose preference test

**FIGURE 7:**
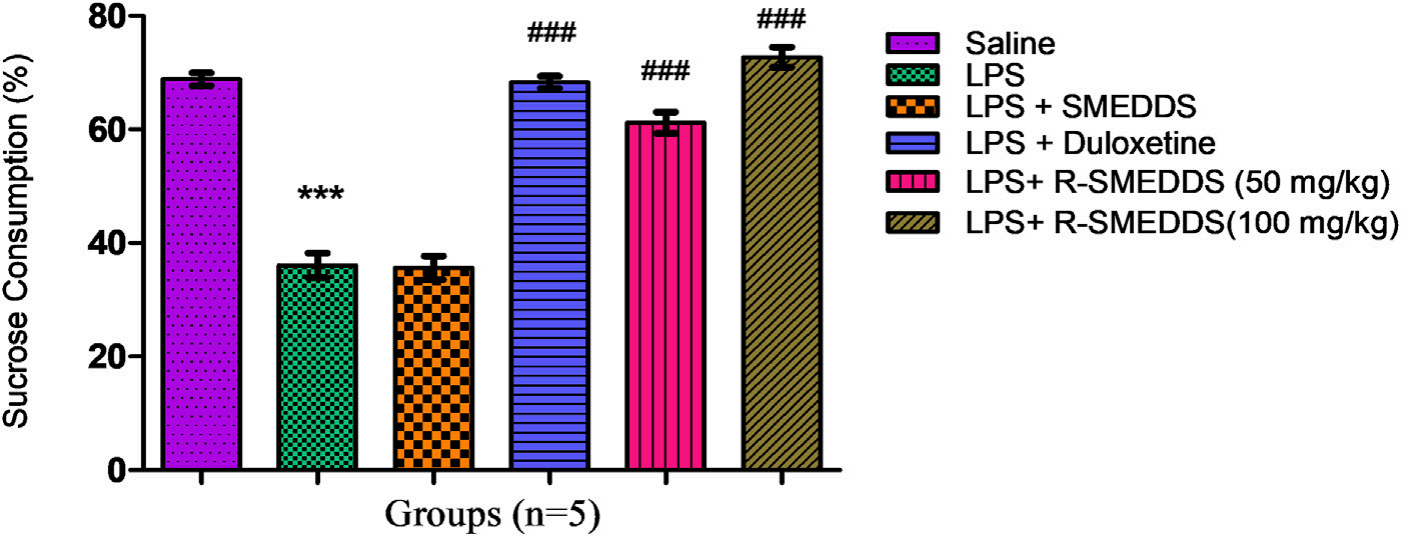
Effects of treatments on sucrose (1% w/v) consumption in an LPS-induced depression model in rats. Results are shown as mean ± SEM (n = 05). ***p < 0.001 compared to the control group; ###p < 0.001 compared to the LPS group.

LPS exposure significantly reduced sucrose preference, indicating anhedonia (a core symptom of depression). Treatment with duloxetine and rhein (50 mg/kg and 100 mg/kg, i.p.) significantly increased the amount of sucrose consumed (68.29 ± 1.044, 61.16 ± 1.865, and 72.73 ± 1.763) compared to the LPS group (36.06 ± 2.189), with both compounds almost completely restoring sucrose preference to control levels (68.85 ± 1.122) [F (5.24) = 94.85, *p* < 0.001)]. The SPT results indicate that both duloxetine and rhein can effectively reverse LPS-induced anhedonia. The effects of rhein on sucrose preference suggest that it could serve as a natural alternative or adjunct to traditional antidepressants, especially for inflammation-related depressive symptoms. The relative percentages of sucrose consumption are outlined in Figure 7.

### 3.7 Biochemical assessment

This study investigates the impact of duloxetine and rhein on the levels of proinflammatory cytokines in rats exposed to LPS, a common model for inducing inflammation-associated depressive-like states. LPS administration is known to elevate cytokine levels such as IL-1β, IL-6, and TNF-α, which contribute to neuroinflammation and are implicated in mood disorders (Naha et al., 2010; Zhao et al., 2019). Both duloxetine and rhein were administered intraperitoneally (i.p.) at 50 mg/kg and 100 mg/kg to examine their anti-inflammatory and neuroprotective properties.

#### 3.7.1 Concentrations of IL-1β

**FIGURE 8:**
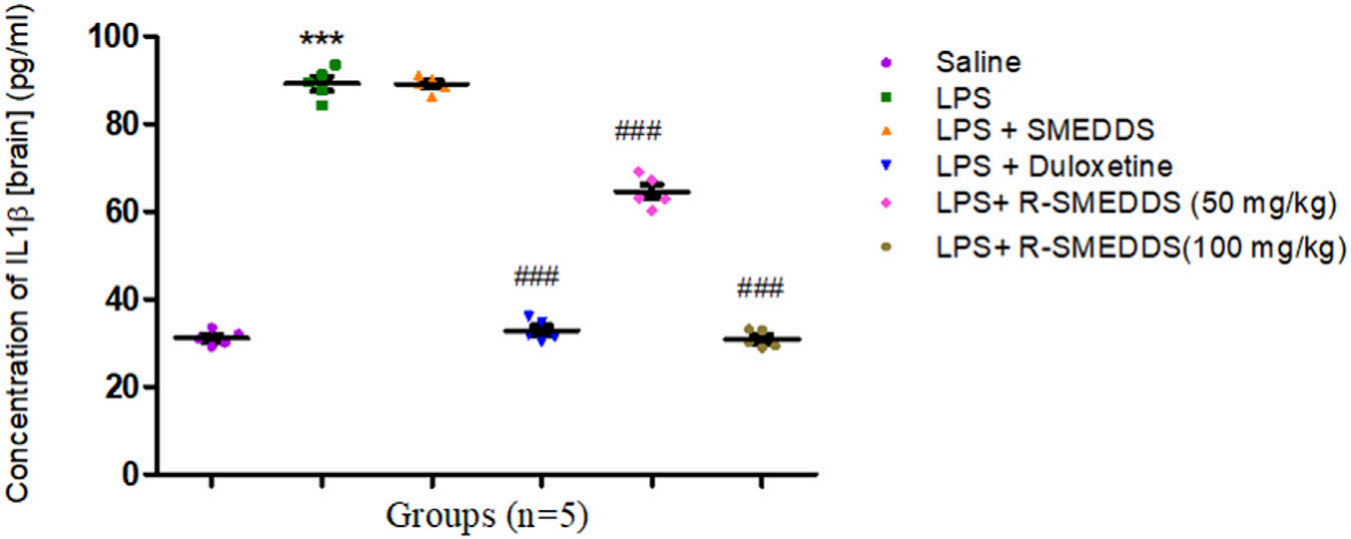
Effects of treatments on IL-1β levels in the brain in an LPS-induced depression model in rats. Results are shown as mean ± SEM (n = 05). ***p < 0.001 compared to the control group; ###p < 0.001 compared to the LPS group.

IL-1β is closely associated with neuroinflammation and synaptic plasticity changes that contribute to depressive symptoms. The observed decrease in IL-1β suggests that both duloxetine and rhein can counteract the neuroinflammatory processes induced by LPS, potentially restoring normal brain function and improving mood-related behaviors (Schmidt et al., 2011). Rhein’s strong effect on IL-1β suggests it may have particular value in treating inflammation-driven depression. LPS administration significantly increased IL-1β levels (89.24 ± 1.571) compared to the saline group (31.19 ± 0.768), indicating a heightened inflammatory response. Both duloxetine and both doses of rhein reduced IL-1β levels (32.86 ± 1.096, 64.51 ± 1.592, and 30.91 ± 0.906) [F (5,24) = 585.3, *p* < 0.001)] with 100 mg/kg (i.p.) of rhein showing a reduction comparable to that seen with 50 mg/kg (i.p.) as outlined in Figure 8.

#### 3.7.2 Concentrations of IL-6

IL-6 is implicated in mood disorders and is a reliable marker of inflammation in the brain. The observed reduction in IL-6 after administration of duloxetine aligns with its antidepressant effects, likely related to lowering stress-related inflammatory responses (Zheng et al., 2020). The ability of rhein to reduce IL-6 levels further highlights its potential anti-inflammatory effects, suggesting a complementary mechanism to traditional antidepressants.

**FIGURE 9:**
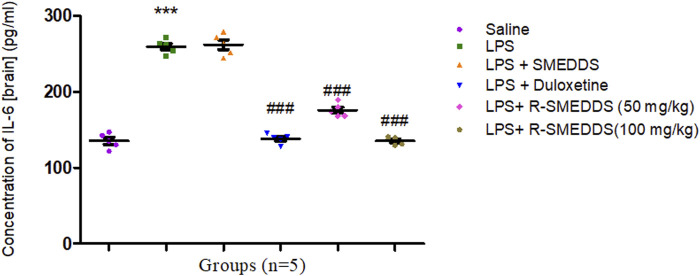
Effects of treatments on interleukin-6 levels in the brain in an LPS-induced depression model in rats. Results are shown as mean ± SEM (n = 05). ***p < 0.001 compared to the control group; ###p < 0.001 compared to the LPS group.

IL-6, another proinflammatory cytokine, was significantly elevated in the LPS group (259.6 ± 4.081) compared to the saline group (135.3 ± 4.445). Both duloxetine and rhein significantly lowered IL-6 levels (138.4 ± 2.984, 175.9 ± 3.977, and 133.9 ± 2.232) [F (5,24) = 211.8, *p* < 0.001)], with a more pronounced reduction at the higher dose of rhein as depicted in Figure 9.

#### 3.7.3 Concentrations of TNF-α

**FIGURE 10:**
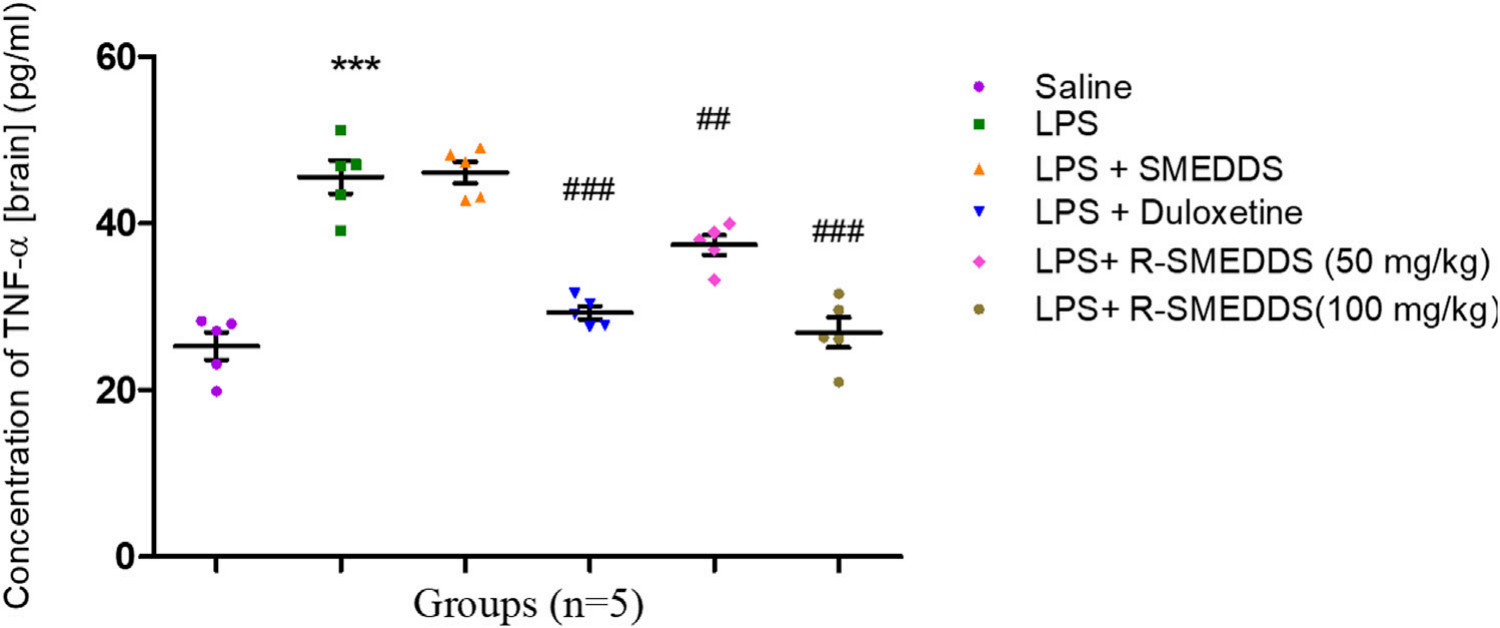
Effects of treatments on TNF-α levels in the brain in an LPS-induced depression model in rats. Results are shown as mean ± SEM (n = 05). ***p < 0.001 compared to the control group; ##p < 0.01, ###p < 0.001 compared to the LPS group.

TNF-α is a key mediator in the inflammatory cascade associated with depressive-like behavior. The reduction in TNF-α levels by both duloxetine and rhein suggests that they may alleviate depressive symptoms by suppressing proinflammatory signaling (Lang and Borgwardt, 2013). The effectiveness of rhein, particularly at the higher dose, likely reflects its direct anti-inflammatory properties, while duloxetine’s impact might result from indirect anti-inflammatory effects through neurotransmitter modulation. LPS administration led to a marked increase in TNF-α levels (45.57 ± 2.012) in the brain homogenate compared to controls (25.27 ± 1.642). Treatment with duloxetine and rhein significantly reduced TNF-α levels in a dose-dependent manner (29.30 ± 0.771, 37.40 ± 1.162 and 26.93 ± 1.807) [F (5.24) = 37.89, *p* < 0.001)], with the 100 mg/kg (i.p.) dose showing the most substantial reduction, as shown in Figure 10.

### 3.8 Effect of rhein on the histologic structure of the hippocampus

The hippocampus comprises the C-shaped cornu ammonis (CA) and the interlocking V-shaped dentate gyrus (DG). The cornu ammonis includes three regions: CA1, CA2, and CA3. This study specifically examined the CA3 region and the DG, as they are reported to be affected in depression.

In all slices assessed for CA3, the CA3 region consisted of polymorphic, pyramidal, and molecular cell layers. The DG regions consisted of a molecular layer (ML), granular cell layer (GCL), and pleomorphic layer (PL).

#### 3.8.1 Saline group (control)

**FIGURE 11:**
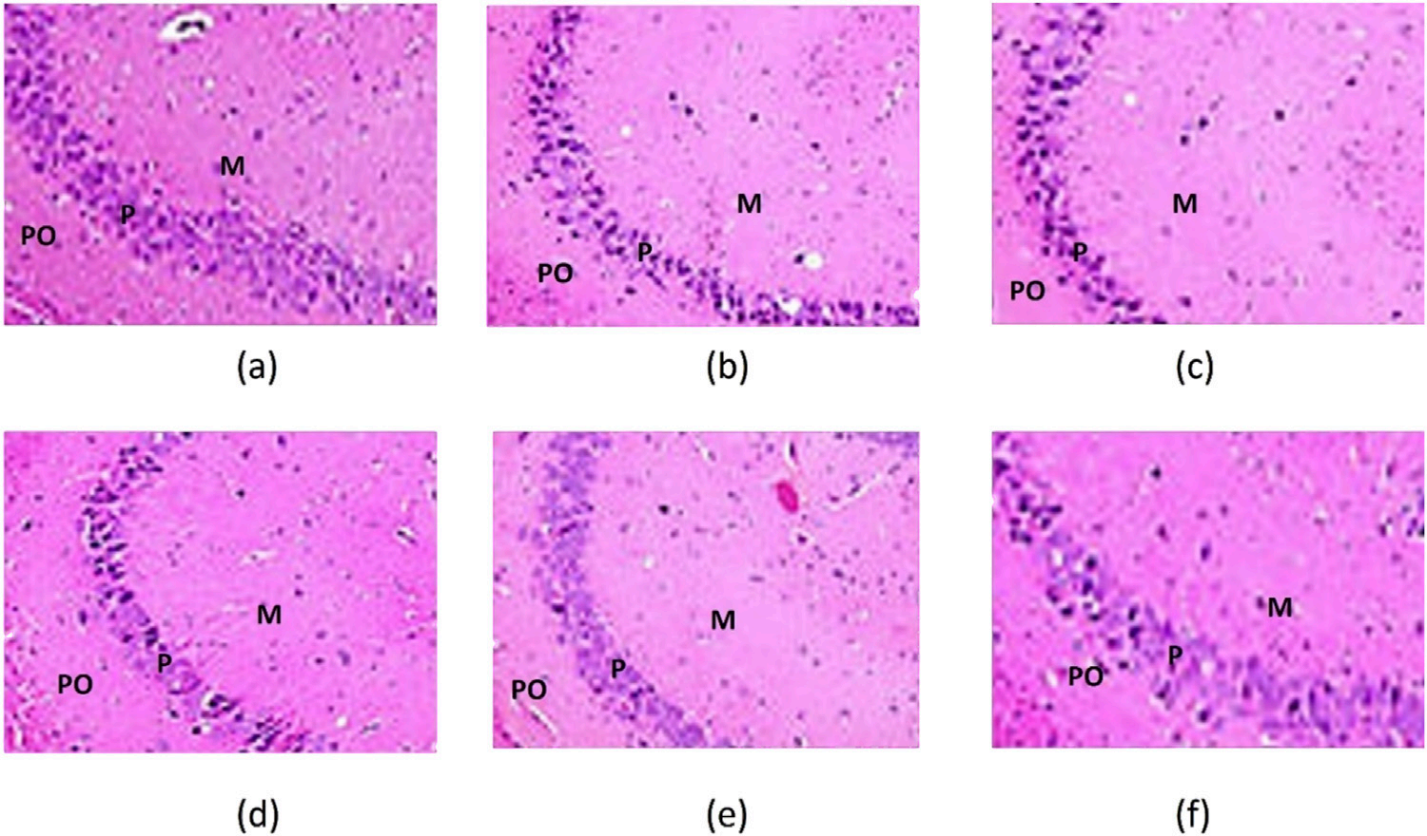
CA3 of the hippocampus of the saline. **(a)** LPS; **(b)** LPS + SNEDDS; **(c)** LPS + Dulo; **(d)** LPS + R-SNEDSS; 50 mg/kg, i.p.; and **(e)** LPS + R-SNEDSS; 100 mg/kg, i.p. **(f)** Groups show three layers; the polymorphic (PO), the pyramidal (P), and the molecular (M). Note the changes in the pyramidal layer thickness. H&E staining. (×400). Dulo: duloxetine.

CA3 region: There was normal neuronal morphology with intact pyramidal neurons. Thus, no signs of neuronal degeneration, gliosis, or vacuolation were observed, as shown in Figure 11a.

**FIGURE 12:**
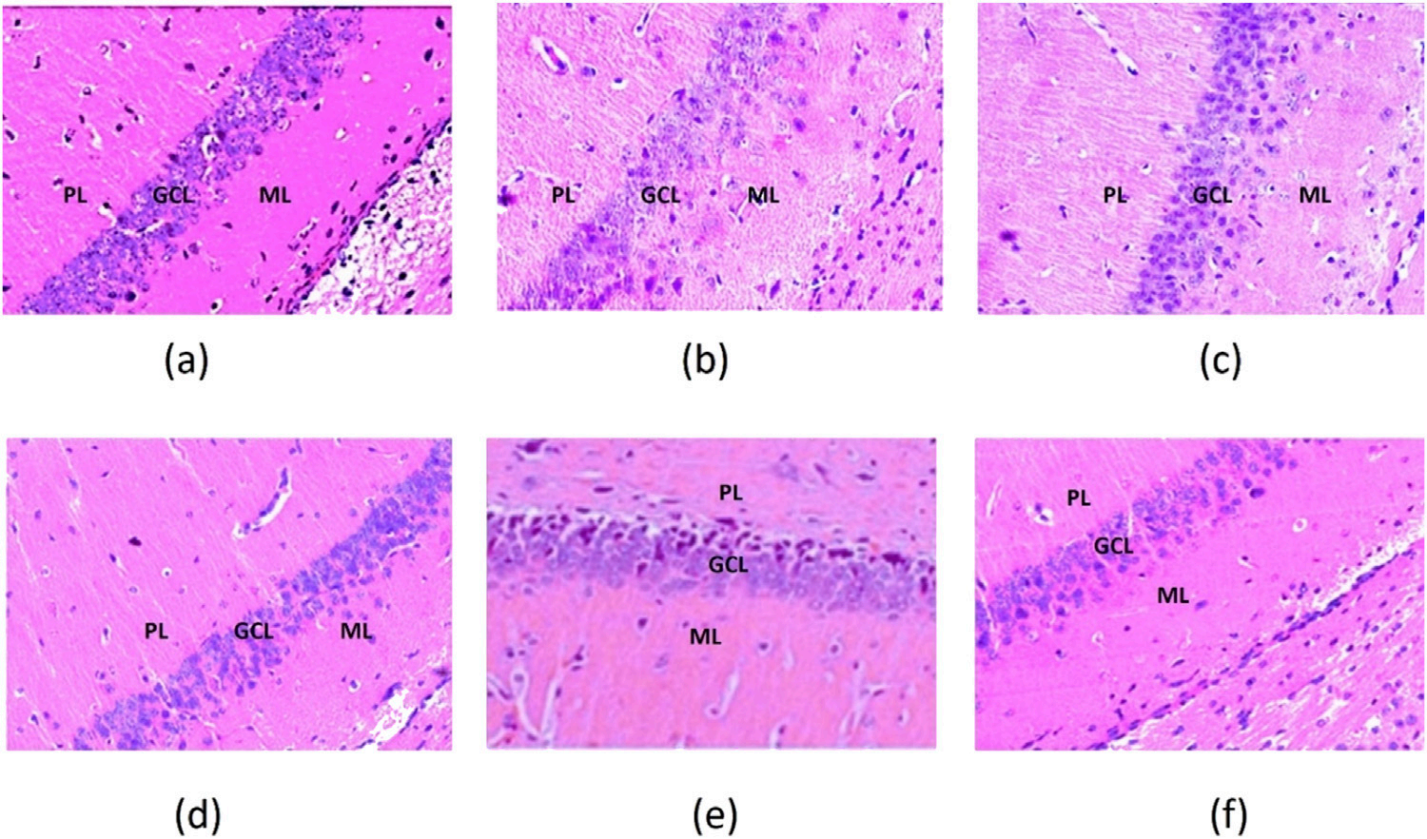
Dentate gyrus of the hippocampus of the saline. **(a)** LPS; **(b)** LPS + SNEDDS; **(c)** LPS + Dulo; **(d)** LPS + R-SNEDSS; 50 mg/kg, i.p.; and **(e)** LPS + R-SNEDSS; 100 mg/kg, i.p. **(f)** Groups show molecular (ML), granular cell (GCL), and pleomorphic layers (PL). Note the intactness of the granular cell layer and neuronal degeneration. H&E staining. (×400). Dulo: duloxetine.

DG region: There was a preserved granule cell layer with no evident morphological abnormalities. Thus, the samples exhibited an absence of inflammatory cell infiltration or necrosis, which is revealed in Figure 12a.

#### 3.8.2 LPS group (lipopolysaccharide-induced neuroinflammation)

**CA3 region:** Marked neuronal degeneration, including pyknotic nuclei and vacuolated cytoplasm, were noted, as were increased microglial activation and gliosis. Therefore, evidence of mild-to-moderate edema, as shown in Figure 11b, was confirmed.

**DG region:** Disruption of granule cell architecture with reduced cellular density demonstrated the presence of apoptotic bodies and increased inflammatory infiltrates, as shown in Figure 12b.

#### 3.8.3 LPS + SNEDDS group

CA3 region: Marked neuronal degeneration, including pyknotic nuclei and vacuolated cytoplasm, were noted, as were increased microglial activation and gliosis, which showed evidence of edema, as shown in Figure 11c.

DG region: Disruption of granule cell architecture with reduced cellular density indicated the presence of apoptotic bodies and increased inflammatory infiltrates, as shown in Figure 12c.

#### 3.8.4 LPS + duloxetine group

CA3 region: Significant neuroprotection with fewer degenerative changes was noted, as were reduced edema and microglial activation, as shown in Figure 11d.

DG region: Near-normal granule cell layer morphology with minimal apoptotic features indicated a markedly decreased inflammatory response, as shown in Figure 12d.

#### 3.8.5 LPS + R-SNEDDS (50 mg/kg, i.p.) group

CA3 region: Samples showed enhanced neuronal preservation compared to the LPS group. Mild gliosis with occasional microglial activation is shown in Figure 11e.

DG region: Near-normal cellular architecture with minimal disruption of the granule cell layer indicated the reduction in inflammatory markers compared to the LPS group, as shown in Figure 12e.

#### 3.8.6 LPS + R-SNEDDS (100 mg/kg, i.p.) group

CA3 region: Prominent neuroprotective effects with almost normal neuronal morphology, minimal gliosis, and no microglial activation are shown in Figure 11f.

DG region: A fully preserved granule cell layer with negligible apoptotic or necrotic changes indicated the absence of significant inflammatory infiltrates, as shown in Figure 12f.

## 4 Discussion

Depression has a multifaceted etiology, and inflammatory mediators have played a crucial role in the etiology of depression (Kennis et al., 2020). Conventional antidepressant therapy marks only a single etiology, the monoamine theory of depression. Furthermore, it has various undesirable side effects, such as sedation, hypotension, weight gain, indigestion, or sexual dysfunction (Bakunina et al., 2015). This leads to poor patient compliance and discontinuation of medication with relapse of depressive symptoms and increased risk (Lopresti, 2019). The evidence suggests that anti-inflammatory drugs may block the manifestation of depression. Additionally, depressed individuals tend to exhibit elevated levels of proinflammatory cytokines, chemokines, and cellular adhesion molecules. Moreover, these proinflammatory cytokines have been shown to influence various pathophysiological aspects of depression, such as neurotransmitter metabolism and neuroendocrine function, increased oxidative stress, increased corticosteroids, neurogenesis, synaptic plasticity, and behavior. Stress can also precipitate depression because it promotes inflammatory responses (Zobel et al., 1999).

It is important to identify an innovative active constituent that effectively targets the complex causes of depression while minimizing side effects. Rhein has demonstrated significant effectiveness in addressing various neurological disorders associated with depression (Wang et al., 2015; Wang et al., 2016). Consequently, we believe that rhein may serve as an effective alternative and complementary therapeutic target for addressing depression (Cryan et al., 2002). This approach promises not only to enhance treatment outcomes but also to unveil previously undiscovered mechanistic insights related to the action of antidepressants. The current study aims to identify new targets for treating depression, specifically in patients experiencing inflammation-associated depressive symptoms, through docking studies that reveal the potential binding of rhein to proinflammatory cytokine target proteins.

The storm of cytokines-mediated interleukins and TNF-α influences the neurodegeneration of adrenergic pathways (Bhatt et al., 2020; Gross and Seroogy, 2020). Bioactive compound rhein showed a potent inhibitory effect against interleukin and necrosis factors. As per the docking result shown in Figure 3, rhein potentially binds to target enzymes 1ALU, 2AZ5, and 5R88 with docking scores of −5.84 kcal/mol, −5.23 kcal/mol, and −5.24 kcal/mol, respectively. Rhein interacts with the 1ALU protein through various bonds with several amino acid residues present in the protein. Gln175 forms a hydrogen bond with its hydroxyl group (-OH), while Arg182 and Arg179 interact with the carboxyl group (-COO^−^). Leu178 and Leu33 contribute through hydrophobic interactions, while Arg30 and Asp34 engage in electrostatic interactions and stabilize the ligand within the binding pocket. Rhein interacts with protein 2AZ5, and in this interaction, Tyr151 forms hydrogen bonds with its -OH, and Tyr59, Leu57, and Ile155 are observed with hydrophobic interactions. Additionally, Gly121 and Gly122 have a role in structural support. The rhein ligand interacts with protein 5S88 through various bonds and interactions, such as Lys74 forming electrostatic interactions with the negatively charged -COO^−^ group of rhein. Tyr24 and Leu82 form hydrogen bonds with the -OH groups of the compound, while Phe133, Pro131, Leu80, and Leu82 form hydrophobic interactions, which stabilize the ligand within the binding site. These interactions collectively ensure the strong binding of rhein within the active site of the proteins. Noteworthy, the bioactive compound rhein has offered an excellent antidepressive action. The presence of a hydroxyl group and a keto function in the rhein structure showed prominent binding with targeted enzymes. The compounds providing an antidepressant effect have the lowest binding energy to the receptor. Therefore, the bioactive compound rhein generated the best conformation of the ligand-receptor complex and has a potential antidepressant effect that may be further enhanced and developed as a lead therapeutic phytoconstituent (Danao et al., 2023).

R-SNEDDS was evaluated for prophylactic antidepressant effect by lipopolysaccharide-induced depression in rats. The study highlights the promising outcomes linked to its anti-inflammatory and antidepressant properties. The docking study outlines the potential affinity of rhein with proinflammatory cytokines associated with neuroinflammation. Immune cells secrete tiny signaling proteins called cytokines, which are essential for regulating immune responses. They play a pivotal role in the pathophysiological alterations in the brain that lead to depression symptoms (Bennett and Molofsky, 2019; Kharwade et al., 2023). In LPS-induced models of depression, an endotoxin challenge activates the immune system through toll-like receptor 4 (TLR4). This activation results in the production of key proinflammatory cytokines, such as TNF-α, IL-1β, and IL-6.

TNF-α is a major cause of inflammation and has been demonstrated to reduce synaptic plasticity by interfering with long-term potentiation (LTP), which is essential for memory and learning (Liu et al., 2017). Elevated TNF-α levels also impair serotonin synthesis by reducing the availability of its precursor, tryptophan, through the activation of the indoleamine 2,3-dioxygenase (IDO) pathway, while our results showed reduced levels of TNF-α in the brain. IL-1β exacerbates neuronal excitotoxicity by increasing glutamate release and reducing glutamate uptake in astrocytes, leading to neuronal damage (Zhang et al., 2024). IL-1β also reduces brain-derived neurotrophic factor (BDNF) expression, impairing neurogenesis in the hippocampus, while our results showed reduced levels of IL-1β in the brain. IL-6 disrupts the blood–brain barrier (BBB), facilitating the entry of peripheral inflammatory molecules into the brain (Rothaug et al., 2016). This cytokine also activates the HPA axis, resulting in hypercortisolemia, a hallmark of chronic stress and depression, while our results showed that it reduced the levels of IL-6 in the brain.

Proinflammatory cytokines also activate microglia, the resident immune cells of the CNS. Activated microglia release additional cytokines, chemokines, and reactive oxygen species (ROS), prolonging a cycle of neuroinflammation (Zhang et al., 2023). Persistent microglial activation alters neuronal connectivity and function by promoting synaptic clipping inappropriately, leading to the loss of synaptic density and inducing oxidative stress and mitochondrial dysfunction, which compromise neuronal survival.

Rhein is an anti-inflammatory active ingredient enriched in rhubarb. It has been found that rhein inhibited IL-1β-induced activation of NF-κB and AP-1 in hypoxic cultured chondrocytes (Bi et al., 2020). Rhein suppresses the activation of NF-κB, a key transcription factor in inflammation. It blocks IκBα phosphorylation and degradation, preventing NF-κB from translocating to the nucleus and reducing the expression of proinflammatory cytokines (TNF-α, IL-6, and IL-1β). This downregulation reduces neuroinflammation, which is linked to depression (Wang et al., 2018). TNF-α and IL-1β increase the expression of IDO, an enzyme that redirects tryptophan metabolism toward the kynurenine pathway. This pathway produces neuroactive metabolites, such as kynurenic acid, which can reduce the availability of serotonin by depleting tryptophan. Additionally, the increased production of neurotoxic kynurenines contributes to the development of depression (Muneer, 2020). Cytokines activate the HPA axis by triggering the release of corticotropin-releasing hormone and adrenocorticotropic hormone. Chronic activation leads to elevated cortisol levels, which damage hippocampal neurons and suppress BDNF expression.

Rhein inhibits p38 MAPK, ERK1/2, and JNK phosphorylation, preventing inflammatory cytokine production. By reducing the MAPK-driven release of TNF-α and IL-6, rhein protects neurons from inflammatory damage. This mechanism is crucial in microglial activation, where MAPK inhibition can reduce neuroinflammatory responses (Wang et al., 2018).

Rhein pretreatment interrupts this pathogenic cascade by reducing the transcription of proinflammatory cytokines (TNF-α, IL-1β, and IL-6). This mitigates systemic and central neuroinflammation. By reducing cytokine levels, rhein may alleviate cytokine-mediated disruptions in serotonin and dopamine pathways. Rhein’s antioxidative properties reduce ROS production, protecting neurons from damage (Yin et al., 2021). Rhein’s ability to inhibit NF-κB and MAPK pathways contributes to its anti-inflammatory and antidepressant effects. The behavioral improvements observed in TST, FST, OFT, and SPT suggest that it reduces depressive symptoms through neuroinflammatory modulation. This makes rhein a promising candidate for novel antidepressant therapies targeting neuroinflammation (Ge et al., 2017).

By modulating inflammation and oxidative stress, rhein may indirectly restore BDNF levels and support hippocampal neurogenesis. Behavioral parameters were assessed through the TST, FST, OFT, and SPT. Results from TST and FST indicated a significant reduction in immobility time, suggesting a decrease in despair-like behavior, while the OFT showed improved exploratory activity, implying reduced anxiety. Additionally, the SPT demonstrated increased sucrose consumption, indicating a reversal of anhedonia (McArthur and Borsini, 2006). Consequently, the study indicates that the R-SNEDDS may have antidepressant effects by modulating TNF-α and interleukin pathways, presenting a promising therapeutic approach for depression associated with inflammation. Our findings suggest that the R-SNEDDS is a potential biomolecule for treating depression.

The histopathological findings of this study demonstrate the protective effects of rhein and duloxetine against hippocampal damage induced by lipopolysaccharides. The hippocampus, particularly the CA3 region and the dentate gyrus (DG), is highly vulnerable to inflammatory insults, which are associated with depressive disorders. Significant histological abnormalities were observed in the CA3 pyramidal cells and DG granular cells in rats treated with LPS. These included reduced cell size, darkened cytoplasm, and condensed nuclei, which are indicative of apoptosis. These changes are consistent with the neuroinflammatory responses commonly seen in models of depression.

Treatment with rhein and duloxetine reduced structural alterations, as shown by the preservation of normal cellular morphology. This suggests that R-SNEDDS (100 mg/kg, i.p.) may have a neuroprotective role, likely due to its anti-inflammatory and antioxidant properties. Hematoxylin and eosin staining allowed for a clear visualization of cellular architecture, facilitating a detailed assessment of the treatment effects. These findings support the potential use of R-SNEDDS as a promising therapeutic target for neuroinflammatory and depressive disorders, highlighting the importance of maintaining hippocampal integrity to support cognitive and emotional health. SNEDDS is a promising approach to enhancing the bioavailability of lipophilic drugs, particularly in the realm of CNS drug delivery. Recent advancements have focused on improving the efficiency of SNEDDS in crossing the BBB and delivering therapeutic agents effectively to the brain.

Recently, researchers developed solid SNEDDS (S-SNEDDS) using chitosan–EDTA microparticles for the delivery of cyclosporine, a BCS class II drug. The study demonstrated that these microparticles exhibit superior adsorbent characteristics, potentially improving drug absorption in the CNS (More S. M. et al., 2025). A study published in 2023 highlighted the potential of nanocarriers, including SNEDDS, in facilitating the transport of phytochemicals across the BBB. These systems enhance drug solubility, stability, and bioavailability, offering a promising strategy for treating neurological disorders (Mishra et al., 2023).

## 5 Conclusion

The molecular link between proinflammatory cytokines and depression involves a complex interplay of pathways that disrupt neurotransmission, neurogenesis, and brain plasticity. The ability of rhein to inhibit proinflammatory cytokine production and regulate neuroinflammatory pathways, combined with improved performance in behavioral tests, highlights its potential as a multifaceted therapeutic agent for inflammation-associated depression. These results also support the broader hypothesis that neuroinflammation plays a significant role in the pathophysiology of depression and that targeting inflammatory pathways offers a viable therapeutic strategy. In conclusion, the present study demonstrated that treatment with R-SNEDDS markedly reduces inflammatory markers compared to the LPS group. It confirmed that R-SNEDDS could have anti-neuroinflammatory properties and is a promising therapeutic candidate for the management of depression. However, future studies are required to investigate the precise molecular mechanisms of antidepressant activity of rhein. This investigation is currently in the initial stages and requires clinical data on higher experimental animals (e.g., rabbits, dogs, and monkeys) to determine its risk-benefit ratio and the safety of participants.

## Data Availability

The original contributions presented in the study are included in the article/supplementary material; further inquiries can be directed to the corresponding authors.

## References

[B1] Ahmad H. Arya A. Agrawal S. Mall P. Samuel S. S. Sharma K. (2016a). Rutin phospholipid complexes confer neuro-protection in ischemic-stroke rats. RSC Adv. 6 (99), 96445–96454. 10.1039/c6ra17874j

[B2] Ahmad H. Arya A. Agrawal S. Samuel S. S. Singh S. K. Valicherla G. R. (2016b). Phospholipid complexation of NMITLI118RT+: way to a prudent therapeutic approach for beneficial outcomes in ischemic stroke in rats. Drug. Deliv. 23 (9), 3606–3618. 10.1080/10717544.2016.1212950 27685355

[B3] Alogheli H. Olanders G. Schaal W. Brandt P. Karlén A. (2017). Docking of macrocycles: comparing rigid and flexible docking in glide. J. Chem. Inf. Model. 57 (2), 190–202. 10.1021/acs.jcim.6b00443 28079375

[B4] Álvarez-González I. Camacho-Cantera S. Gómez-González P. Barrón M. J. R. Morales-González J. A. Madrigal-Santillán E. O. (2021). Genotoxic and oxidative effect of duloxetine on mouse brain and liver tissues. Sci. Rep. 11 (1), 6897. 10.1038/s41598-021-86366-0 33767322 PMC7994804

[B5] Bai Y. Cai Y. Chang D. Li D. Huo X. Zhu T. (2024). Immunotherapy for depression: recent insights and future targets. Pharmacol. Ther. 257, 108624. 10.1016/j.pharmthera.2024.108624 38442780

[B6] Bakunina N. Pariante C. M. Zunszain P. A. (2015). Immune mechanisms linked to depression via oxidative stress and neuroprogression. Immunol 144, 365–373. 10.1111/imm.12443 PMC4557673 25580634

[B7] Baloch J. Sohail M. F. Sarwar H. S. Kiani M. H. Khan G. M. Jahan S. (2019). Self-nanoemulsifying drug delivery system (Snedds) for improved oral bioavailability of chlorpromazine: *in vitro* and *in vivo* evaluation. Med 55 (5), 210. 10.3390/medicina55050210 PMC6572212 31137751

[B8] Bancroft J. D. Gamble M. (2007). Theory and practice of histological techniques. Sixth Edition. Elsevier. 10.1097/nen.0b013e31817e2933

[B9] Barua C. C. Haloi P. Saikia B. Sulakhiya K. Pathak D. C. Tamuli S. (2018). Zanthoxylum alatum abrogates lipopolysaccharide-induced depression-like behaviours in mice by modulating neuroinflammation and monoamine neurotransmitters in the hippocampus. Pharm. Biol. 56, 245–252. 10.1080/13880209.2017.1391298 29569964 PMC6130615

[B10] Bathula R. (2022). Glide docking, autodock, binding free energy and drug- likeness studies for prediction of potential inhibitors of cyclin-dependent kinase 14 protein in wnt signaling pathway. Biointerface Res. Appl. Chem. 12 (2), 2473–2488. 10.33263/BRIAC122.24732488

[B11] Bennett F. C. Molofsky A. V. (2019). The immune system and psychiatric disease: a basic science perspective. Clin. Exp. Immunol. 197 (3), 294–307. 10.1111/cei.13334 31125426 PMC6693968

[B12] Bhatt S. Nagappa A. N. Patil C. R. (2020). Role of oxidative stress in depression. Drug Discov. Today. 25, 1270–1276. 10.1016/j.drudis.2020.05.001 32404275

[B13] Bi F. Ma H. Ji C. Chang C. Liu W. Xie K. (2020). Rhein protects against neurological deficits after traumatic brain injury in mice via inhibiting neuronal pyroptosis. Front. Pharmacol. 11, 564367–564368. 10.3389/fphar.2020.564367 33101024 PMC7554525

[B14] Bosker F. J. Westerink B. H. C. Cremers T. I. F. H. Gerrits M. Van Der Hart M. G. C. Kuipers S. D. (2004). Future antidepressants: what is in the pipeline and what is missing? CNS. Drugs. 18, 705–732. 10.2165/00023210-200418110-00002 15330686

[B15] Burstein O. Doron R. (2018). The unpredictable chronic mild stress protocol for inducing anhedonia in mice. J. Vis. Exp. 140, 58184. 10.3791/58184 PMC6235587 30417885

[B16] Cryan J. F. Markou A. Lucki I. (2002). Assessing antidepressant activity in rodents: recent developments and future needs. Trends. Pharmacol. Sci. 23 (5), 238–245. 10.1016/S0165-6147(02)02017-5 12008002

[B17] Daina A. Michielin O. Zoete V. (2017). SwissADME: a free web tool to evaluate pharmacokinetics, drug-likeness and medicinal chemistry friendliness of small molecules. Sci. Rep. 7, 42717. 10.1038/srep42717 28256516 PMC5335600

[B18] Danao K. Kale S. Rokde V. Nandurkar D. Mahajan U. Dumore N. (2023). *In silico* prediction of antidiabetic activity of phytoconstituents of pterocarpus marsupium targeting α-amylase enzyme. Biosci. Biotechnol. Res. Asia 20, 147–162. 10.13005/bbra/3077

[B19] Domingues M. Casaril A. M. Birmann P. T. Lourenço D. de A. Vieira B. Begnini K. (2018). Selanylimidazopyridine prevents lipopolysaccharide-induced depressive-like behavior in mice by targeting neurotrophins and inflammatory/oxidative mediators. Front. Neurosci. 12, 486–510. 10.3389/fnins.2018.00486 30072867 PMC6060445

[B20] Duman R. S. (2004). Role of neurotrophic factors in the etiology and treatment of mood disorders. NeuroMolecular. Med. 5, 11–25. 10.1385/nmm:5:1:011 15001809

[B21] Fanelli D. Weller G. Liu H. (2021). New serotonin-norepinephrine reuptake inhibitors and their anesthetic and analgesic considerations. Neurol. Int. 13, 497–509. 10.3390/neurolint13040049 34698218 PMC8544373

[B22] Ge H. Tang H. Liang Y. Wu J. Yang Q. Zeng L. (2017). Rhein attenuates inflammation through inhibition of NF-κB and NALP3 inflammasome *in vivo* and *in vitro* . Drug Des. devel. Ther. 11, 1663–1671. 10.2147/DDDT.S133069 PMC5472410 28652704

[B23] Gross C. Seroogy K. B. (2020). Neuroprotective roles of neurotrophic factors in depression. Elsevier Inc. 10.1016/B978-0-12-814037-6.00007-0

[B24] Kaleem M. Haque S. E. (2015). Evaluation of cardioprotective role of vinpocetine in isoproterenolinduced myocardial infarction in rats. J. Pharm. Res. 9, 408–414. Available online at: https://www.researchgate.net/publication/342480421_Evaluation_of_Cardioprotective_role_of_Vinpocetine_in_Isoproterenol-induced_Myocardial_Infarction_in_Rats (Accessed November 29, 2021).

[B25] Kennis M. Gerritsen L. van Dalen M. Williams A. Cuijpers P. Bockting C. (2020). Prospective biomarkers of major depressive disorder: a systematic review and meta-analysis. Mol. Psychiatry. 25, 321–338. 10.1038/s41380-019-0585-z 31745238 PMC6974432

[B26] Kharwade R. Kazi M. Mahajan N. Badole P. More S. Kayali A. (2024). Mannosylated PAMAM G2 dendrimers mediated rate programmed delivery of efavirenz target HIV viral latency at reservoirs. Saudi Pharm. J. 32, 102154. 10.1016/j.jsps.2024.102154 39282004 PMC11399684

[B27] Kharwade R. Mahajan N. More S. Warokar A. Dhobley A. Palve D. (2023). Effect of PEGylation on drug uptake, biodistribution, and tissue toxicity of efavirenz – ritonavir loaded PAMAM G4 dendrimers. Pharm. Dev. Technol. 28 (2), 200–218. 10.1080/10837450.2023.2173230 36695103

[B28] Kharwade R. More S. Suresh E. Warokar A. Mahajan N. Mahajan U. (2022). Improvement in bioavailability and pharmacokinetic characteristics of efavirenz with booster dose of ritonavir in PEGylated PAMAM G4 dendrimers. AAPS PharmSciTech 23 (6), 177. 10.1208/s12249-022-02315-8 35750994

[B29] Kharwade R. S. More S. M. (2021). Coordinated roadmap to grip pandemic COVID-19. Coronaviruses 2, 468–480. 10.2174/2666796701999200801023110

[B30] Koch A. E. Kunkel S. L. Harlow L. A. Johnson B. Evanoff H. L. Haines G. K. (1992). Enhanced production of monocyte chemoattractant protein-1 in rheumatoid arthritis. J. Clin. Invest. 90, 772–779. 10.1172/JCI115950 1522232 PMC329929

[B31] Kumar Tiwari P. Chouhan M. Mishra R. Gupta S. Chaudhary A. A. Al-Zharani M. (2024). Structure-based virtual screening methods for the identification of novel phytochemical inhibitors targeting furin protease for the management of COVID-19. Front. Cell. Infect. Microbiol. 14, 1391288–1391313. 10.3389/fcimb.2024.1391288 38919703 PMC11196402

[B32] Laddha P. Suthar V. Butani S. (2014). Development and optimization of self microemulsifying drug delivery of domperidone. Braz. J. Pharm. Sci. 50, 91–100. 10.1590/S1984-82502011000100009

[B33] Lang U. E. Borgwardt S. (2013). Molecular mechanisms of depression: perspectives on new treatment strategies. Cell. Physiol. biochem. 31, 761–777. 10.1159/000350094 23735822

[B34] Li M. Fu Q. Li Y. Li S. Xue J. Ma S. (2014). Emodin opposes chronic unpredictable mild stress induced depressive-like behavior in mice by upregulating the levels of hippocampal glucocorticoid receptor and brain-derived neurotrophic factor. Fitoterapia 98, 1–10. 10.1016/j.fitote.2014.06.007 24932776

[B35] Li X. Chu S. Liu Y. Chen N. (2019). Neuroprotective effects of anthraquinones from rhubarb in central nervous system diseases. *Evidence-Based* . Complement. Altern. Med. 2019, 3790728. 10.1155/2019/3790728 PMC6541978 31223328

[B36] Liu M. Y. Yin C. Y. Zhu L. J. Zhu X. H. Xu C. Luo C. X. (2018). Sucrose preference test for measurement of stress-induced anhedonia in mice. Nat. Protoc. 13, 1686–1698. 10.1038/s41596-018-0011-z 29988104

[B37] Liu Y. Zhou L. J. Wang J. Li D. Ren W. J. Peng J. (2017). TNF-α differentially regulates synaptic plasticity in the hippocampus and spinal cord by microglia-dependent mechanisms after peripheral nerve injury. J. Neurosci. 37 (4), 871–881. 10.1523/JNEUROSCI.2235-16.2016 28123022 PMC5296781

[B38] Lopresti A. L. (2019). Mitochondrial dysfunction and oxidative stress: relevance to the pathophysiology and treatment of depression. Elsevier Inc. 10.1016/B978-0-12-813333-0.00015-9

[B39] Madhavi Sastry G. Adzhigirey M. Day T. Annabhimoju R. Sherman W. (2013). Protein and ligand preparation: parameters, protocols, and influence on virtual screening enrichments. J. Comput. Aided. Mol. Des. 27, 221–234. 10.1007/s10822-013-9644-8 23579614

[B40] Massart R. Mongeau R. Lanfumey L. (2012). Beyond the monoaminergic hypothesis: neuroplasticity and epigenetic changes in a transgenic mouse model of depression. Philos. Trans. R. Soc. B Biol. Sci. 367, 2485–2494. 10.1098/rstb.2012.0212 PMC3405682 22826347

[B41] McArthur R. Borsini F. (2006). Animal models of depression in drug discovery: a historical perspective. Pharmacol. Biochem. Behav. 84, 436–452. 10.1016/j.pbb.2006.06.005 16844210

[B42] Mishra K. Rana R. Tripathi S. Siddiqui S. Yadav P. K. Yadav P. N. (2023). Recent advancements in nanocarrier-assisted brain delivery of phytochemicals against neurological diseases. Neurochem. Res. 48 (10), 2936–2968. 10.1007/s11064-023-03955-3 37278860

[B43] More S. Kaleem M. Kharwade R. Almutairy A. F. Shahzad N. Ali Mujtaba M. (2025a). Depression unveiled: insights into etiology and animal models for behavioral assessment, exploring the multifactorial nature and treatment of depression. Brain. Res. 1847, 149313. 10.1016/j.brainres.2024.149313 39515744

[B44] More S. M. Rashid M. A. Kharwade R. S. Taha M. Alhamhoom Y. Elhassan G. O. (2025b). Development of solid self-nanoemulsifying drug delivery system of rhein to improve biopharmaceutical performance: physiochemical characterization, and pharmacokinetic evaluation. Int. J. Nanomedicine. 20, 267–291. 10.2147/IJN.S499024 39802374 PMC11724663

[B45] Moreau M. Lestage J. Castanon N. Kelley K. W. Castanon N. (2009). Lipopolysaccharide-induced depressive-like behavior is mediated by indoleamine 2, 3-dioxygenase activation in mice. Mol. Psychiatry. 14 (5), 511–522. 10.1038/sj.mp.4002148 18195714 PMC2683474

[B46] Muneer A. (2020). Kynurenine pathway of tryptophan metabolism in neuropsychiatric disorders: pathophysiologic and therapeutic considerations. Clin. Psychopharmacol. Neurosci. 18 (4), 507–526. 10.9758/CPN.2020.18.4.507 33124585 PMC7609208

[B47] Naha P. C. Davoren M. Lyng F. M. Byrne H. J. (2010). Reactive oxygen species (ROS) induced cytokine production and cytotoxicity of PAMAM dendrimers in J774A.1 cells. Toxicol. Appl. Pharmacol. 246, 91–99. 10.1016/j.taap.2010.04.014 20420846

[B48] Naoi M. Shamoto-Nagai M. Maruyama W. (2019). Neuroprotection of multifunctional phytochemicals as novel therapeutic strategy for neurodegenerative disorders: antiapoptotic and antiamyloidogenic activities by modulation of cellular signal pathways. Future Neurol. 14 (1), FNL9. 10.2217/fnl-2018-0028

[B49] Ohgi Y. Futamura T. Kikuchi T. Hashimoto K. (2013). Effects of antidepressants on alternations in serum cytokines and depressive-like behavior in mice after lipopolysaccharide administration. Pharmacol. Biochem. Behav. 103, 853–859. 10.1016/j.pbb.2012.12.003 23262300

[B50] Rajagopal K. Arumugasamy P. Byran G. (2019). *In-silico* drug design, ADMET screening, MM-GBSA binding free energy of some chalcone substituted 9-anilinoacridines as HER2 inhibitors for breast cancer. Int. J. Comput. Theor. Chem. 7 (1), 6. 10.11648/j.ijctc.20190701.12

[B51] Rein T. Ambrée O. Fries G. R. Rappeneau V. Schmidt U. Touma C. (2019). The hypothalamic-pituitary-adrenal axis in depression: molecular regulation, pathophysiological role, and translational implications. Neurobiol. Depress. Road Nov. Ther, 89–96. 10.1016/B978-0-12-813333-0.00009-3

[B52] Rothaug M. Becker-Pauly C. Rose-John S. (2016). The role of interleukin-6 signaling in nervous tissue. Biochim. Biophys. Acta - Mol. Cell Res. 1863, 1218–1227. 10.1016/j.bbamcr.2016.03.018 27016501

[B53] Salokangas R. K. R. Vaahtera K. Pacriev S. Sohlman B. Lehtinen V. (2002). Gender differences in depressive symptoms: an artefact caused by measurement instruments? J. Affect. Disord. 68, 215–220. 10.1016/S0165-0327(00)00315-3 12063149

[B54] Schmidt H. D. Shelton R. C. Duman R. S. (2011). Functional biomarkers of depression: diagnosis, treatment, and pathophysiology. Neuropsychopharmacol 36, 2375–2394. 10.1038/npp.2011.151 PMC3194084 21814182

[B55] Tomaz V. de S. Chaves Filho A. J. M. Cordeiro R. C. Jucá P. M. Soares M. V. R. Barroso P. N. (2020). Antidepressants of different classes cause distinct behavioral and brain pro- and anti-inflammatory changes in mice submitted to an inflammatory model of depression. J. Affect. Disord. 268, 188–200. 10.1016/j.jad.2020.03.022 32174477

[B56] Wang Q. W. Su Y. Sheng J. T. Gu L. M. Zhao Y. Chen X. X. (2018). Anti-influenza a virus activity of rhein through regulating oxidative stress, TLR4, Akt, MAPK, and NF-κB signal pathways. PLoS. One. 13 (1), e0191793. 10.1371/journal.pone.0191793 29385192 PMC5791991

[B57] Wang Y. Fan R. Luo J. Tang T. Xing Z. Xia Z. (2015). An ultra high performance liquid chromatography with tandem mass spectrometry method for plasma and cerebrospinal fluid pharmacokinetics of rhein in patients with traumatic brain injury after administration of rhubarb decoction. J. Sep. Sci. 38, 1100–1108. 10.1002/jssc.201401197 25598181

[B58] Wang Y. Fan X. Tang T. Fan R. Zhang C. Huang Z. (2016). Rhein and rhubarb similarly protect the blood-brain barrier after experimental traumatic brain injury via gp91phox subunit of NADPH oxidase/ROS/ERK/MMP-9 signaling pathway. Sci. Rep. 6, 37098–37113. 10.1038/srep37098 27901023 PMC5128794

[B59] Willner P. (1997). Validity, reliability and utility of the chronic mild stress model of depression: a 10-year review and evaluation. Psychopharmacol(Berl). 134, 319–329. 10.1007/s002130050456 9452163

[B60] Yin R. Zhang K. Li Y. Tang Z. Guo P. Li G. (2023). Lipopolysaccharide-induced depression-like model in mice: meta-analysis and systematic evaluation. Front. Immunol. 14, 1181973. 10.3389/fimmu.2023.1181973 37359525 PMC10285697

[B61] Yin Z. Geng X. Zhang Z. Wang Y. Gao X. (2021). Rhein relieves oxidative stress in an aβ1-42 oligomer-burdened neuron model by activating the SIRT1/PGC-1α-regulated mitochondrial biogenesis. Front. Pharmacol. 12, 746711. 10.3389/fphar.2021.746711 34566664 PMC8461019

[B62] Zeldetz V. Natanel D. Boyko M. Zlotnik A. Shiyntum H. N. Grinshpun J. (2018). A new method for inducing a depression-like behavior in rats 3. Proced. Establishing Depression-Contagion Naïve Rats 12, 746711. 10.3791/57137 PMC5931356 29553503

[B63] Zhang N. Song Y. Wang H. Li X. Lyu Y. Liu J. (2024). IL-1β promotes glutamate excitotoxicity: indications for the link between inflammatory and synaptic vesicle cycle in Ménière’s disease. Cell. death. Discov. 10, 476–511. 10.1038/s41420-024-02246-2 39567494 PMC11579495

[B64] Zhang W. Xiao D. Mao Q. Xia H. (2023). Role of neuroinflammation in neurodegeneration development. Signal Transduct. Target. Ther. 8 (1), 267. 10.1038/s41392-023-01486-5 37433768 PMC10336149

[B65] Zhao X. Cao F. Liu Q. Li X. Xu G. Liu G. (2019). Behavioral, inflammatory and neurochemical disturbances in LPS and UCMS-induced mouse models of depression. Behav. Brain Res. 364, 494–502. 10.1016/j.bbr.2017.05.064 28572058

[B66] Zheng P. Tian X. Zhang W. Yang Z. Zhou J. Zheng J. (2020). Rhein suppresses neuroinflammation via multiple signaling pathways in LPS-stimulated BV2 microglia cells. Evidence-based Complement. Altern. Med. 1, 7210627. 10.1155/2020/7210627 PMC7341424 32714414

[B67] Zobel A. W. Yassouridis A. Frieboes R. M. Holsboer F. (1999). Prediction of medium-term outcome by cortisol response to the combined dexamethasone-CRH test in patients with remitted depression. Am. J. Psychiatry. 156, 949–951. 10.1176/ajp.156.6.949 10360139

